# Multimodal Molecular Imaging: Current Status and Future Directions

**DOI:** 10.1155/2018/1382183

**Published:** 2018-06-05

**Authors:** Min Wu, Jian Shu

**Affiliations:** Department of Radiology, The Affiliated Hospital of Southwest Medical University, Luzhou, Sichuan, China

## Abstract

Molecular imaging has emerged at the end of the last century as an interdisciplinary method involving *in vivo* imaging and molecular biology aiming at identifying living biological processes at a cellular and molecular level in a noninvasive manner. It has a profound role in determining disease changes and facilitating drug research and development, thus creating new medical modalities to monitor human health. At present, a variety of different molecular imaging techniques have their advantages, disadvantages, and limitations. In order to overcome these shortcomings, researchers combine two or more detection techniques to create a new imaging mode, such as multimodal molecular imaging, to obtain a better result and more information regarding monitoring, diagnosis, and treatment. In this review, we first describe the classic molecular imaging technology and its key advantages, and then, we offer some of the latest multimodal molecular imaging modes. Finally, we summarize the great challenges, the future development, and the great potential in this field.

## 1. Introduction

Molecular imaging has emerged at the end of the last century and consists of a combination of in vivo imaging and molecular biology aiming at identifying or describing living biological process at a cellular and molecular level using noninvasive procedures. It is especially addressed to reveal abnormalities in cells and molecules which cause the disease, rather than the final anatomical and structural abnormality caused by cellular or molecular changes [1]. Various modern imaging technologies have been widely used to monitor structural, functional, and molecular changes in cancer tissues, including optical imaging (either by bioluminescence or fluorescence) [2], computed tomography (CT) [3], magnetic resonance imaging (MRI) [4], positron emission tomography (PET) [5], single-photon emission computed tomography (SPECT) [6], and ultrasound (US) [7]. Molecular imaging can detect lesions and determine the nature of earlier lesions more accurately compared to conventional imaging, so that clinicians can effectively intervene in the occurrence and formation stage of the disease [8]. In recent years, with the appearance and development of molecular imaging, early tumor diagnosis became possible. Consequently, the latest advances in multimodal molecular imaging are reviewed in this paper.

In clinical research, CT, MRI, PET, SPECT, US, and optical imaging are usually among the choices of the imaging modalities. Each imaging modality has its own unique strength and intrinsic limitations, such as spatial/depth resolution and sensitivity, making the achievement of precise and reliable information at the disease site difficult. In order to compensate these weak aspects, multimodal molecular imaging has been considered in recent years [9]. Multimodal molecular imaging can play important roles in the clinical care of various diseases by improving clinicians' ability to perform screening, surveillance, staging, prognosis, planning and therapy guidance, monitoring therapy efficacy, and assessing recurrence. With its rapid technological advances, presymptomatic detection, targeted therapy, and personalized medicine may be possible in the near future through the use of multimodal molecular imaging.

## 2. Molecular Imaging Models

At present, molecular imaging models mainly include CT, MRI, radionuclide, ultrasound, and optical imaging.

### 2.1. CT Molecular Imaging

CT is a technique producing images reflecting human anatomy, thanks to differential levels of X-ray attenuation by tissues within the body. CT is widely available, and it has some advantages such as high spatial resolution, strong penetration depth, fast acquisition time, low cost, clinical utility, and relative simplicity. However, CT also has some disadvantages: one is the high radiation dose, which often limits the scan time, and another is the low-quality soft tissue contrast, compared with MRI [10]. In addition, there is the low sensitivity of current CT contrast agents. It is indeed valuable in identifying and assessing several diseases including tumors, brain injury, and pulmonary embolism.

The application of CT molecular imaging requires high quality imaging agents, after injection, in order to achieve the target site for a change in X-ray attenuation. Currently, most CT molecular contrast agents are designed to combine a maximum number of X-ray-absorbing atoms with a nanoparticle, which includes emulsion, liposomes, lipoproteins, and polymeric nanoparticles [11–17].

Until now, multiple examples of the abovementioned CT molecular imaging have started to appear, and there has been a major development in CT molecular imaging. In 2006, Rabin et al. [18] first reported the synthesis of a polymer-coated bismuth sulfate (Bi_2_ S_3_) nanoparticle as an injectable CT imaging agent, which is used for enhanced in vivo imaging of the vasculature, the liver, and lymph nodes in normal mice. Next, Hyafil et al. [19] reported cellular imaging using CT in atherosclerotic plaques in rabbits using an iodinated nanoparticle dispersed with the surfactant. Li et al. [20] reported the use of a 2-deoxy-d-glucose- (2-DG-) labeled gold nanoparticle (AuNP-2-DG) for targeted molecular CT imaging in malignant neoplasms to obtain high-resolution metabolic and anatomic information. Kim et al. [21] reported the synthesis of CT-compatible gold nanoparticles optimized with prostate-specific membrane antigen RNA aptamers and the use of CT molecular imaging and therapy of prostate cancer. Furthermore, Kayyali et al. [22] reported the use of targeted gold nanoparticles and liposomal iodine, respectively, as contrast agents for inner ear imaging, and the results showed that significant enhancement of micro-CT images was observed using liposome iodine. Choi et al. [23] reported the preparation of X-ray CT/US dual-modal imaging probe (GC-DTA-PFP NPs), and the study indicated that X-ray CT/US dual-modal imaging could provide more comprehensive and accurate diagnostic information about the diagnosis of tumor. Yue et al. [24] developed a pH-responsive multifunctional nanotheranostic agent (FePt/GOCNs) for potential in vivo and in vitro dual-modal MRI/CT imaging and in situ cancer inhibition.

The applied research of CT molecular imaging is becoming more and more widespread, and the reason is that they have the potential for dose reduction as low as possible while enhancing image contrast [25], display an X-ray attenuation property better than commercial iodinated small-molecular-contrast agents [26], show much stronger CT imaging effect compared with the traditional small molecule contrast agents [27], and have CT cell-tracking applications for noninvasive monitoring [3, 28, 29]. In short, although CT molecular imaging abilities have not yet been completely explored, it remains an extremely useful morphological tool. When associated with other imaging modalities, CT also can give an anatomical reference frame for the biochemical and physiological findings that are afforded by these other imaging instruments [30].

### 2.2. MRI Molecular Imaging

Today, MRI is seen as the most useful imaging modality in radiology, especially in the detection and characterization of soft tissue pathology [31, 32]. MRI can provide three-dimensional clear images, it has high spatial resolution and high contrast, and the acquisition time and the quality of images are constantly improving, thanks to technological innovations [32]. These aspects are described below by some specific MRI methods.

From a great many studies, including work in packed cells, it is easy to see that cell density varying inversely is reflected in conventional measurements of diffusion using MRI report values of ADC. Consequently, DWI has been used to evaluate tumor cellularity [33, 34]. A quantitative map that acquired tumor cellularity in vivo may be a useful tool for both treatment planning and monitoring. Early studies revealed abnormal water diffusion in various tumors, and more detailed quantitative relationships have always been explored recently between microstructure and ADC. Zhao et al. [35] discovered the ADC changes in response to tumor treatment by measuring water ADC in excised RIF-1 tumors after treatment with an anticancer drug. These data indicated that ADC increase began while the tumors were still growing and implied that it could be an early indicator of valuable cytotoxic treatment response. Hereafter, Henning et al. [36] reported the value of ADC for quantitative assessments of individual tissue regions, tumor growth kinetics, and cell kill in RIF-1 tumor animal models. Moreover, in the related studies, higher pretreatment ADC values incline to correlate with poorer response to therapy and prognosis [37–39]. For example, one of these studies shows that, on performing DWI in hepatic metastases, it appears that ADC values are effective on predicting and monitoring the early chemotherapeutic response of these metastases which originated from gastrointestinal tumors [39]. In these patients, the pretherapy mean ADC value in nonresponding lesions is significantly higher than that in responding lesions. Similarly, Koh et al. [38] found that higher pretreatment ADC values were predictive of poor response to chemotherapy in a small group of patients with colorectal hepatic metastases.

The examples above demonstrate the usefulness of indirect approaches to measure molecular changes in tissues, but many other researchers preferred the approach of a direct molecular imaging using MRI. At present, there are two major types of MRI molecular imaging probes: one is the direct detection of a nuclear species, that is, a component of an imaging probe (e.g., nuclei detection with the use of ^31^P, ^23^Na, ^19^F, ^1^H, or ^13^C within molecules introduced into the body), which is realized by magnetic resonance imaging (MRS), and the other approach consists of an indirect detection via the effects of an agent on the large signal originated from the hydrogen nuclei (protons) in tissue water, either by changing the water relaxation rate or by introducing new pathways for magnetization transfer [40].

For the first of direct approaches, MRS has been widely used to detect metabolic changes in cancerous as well as in normal tissues. MRS can not only provide information on biochemical changes in response to tumor growth but also delineate different metabolic tumor phenotypes. For instance, ^1^H MRS is widely used to monitor metabolic changes in cancer tissue [41–47], and the other active nuclei such as ^31^P (phosphorus) [48, 49], ^23^Na (sodium) [50], ^13^C (carbon) [51–53], and ^19^F (fluorine) [54, 55] are also being used to monitor bioenergetics and metabolic in cancer.

For the second of direct approaches, MRI implies the use of paramagnetic or superparamagnetic agents that alter the tissue proton relaxation time T1, T2, or T2^∗^ or manipulate the magnitude of the water signal via specially designed radiofrequency irradiation that labels one species of protons that in turn transfers the label to the water via magnetization exchange [56]. For example, Ta et al. [57] reported the development of functional multimodal iron oxide nanoparticles for targeted MRI in atherosclerosis, which uses a combination of chemical and biological conjugation techniques. The ultrasmall magnetic dual-contrast iron oxide nanoparticles are used to be efficient positive and negative dual-contrast agents for magnetic resonance imaging and are also labeled with fluorescent molecules to allow for optical imaging.

So far, the design of MR contrast agents may be divided into four different types for different applications. The first is represented by nonspecific contrast agents. For example, the commonly used lanthanide chelates or intravascular blood pool agents [58] are both missing the ability to reach specific targets. Low-molecular-weight Gd(III) complexes have become an essential tool in the detection and characterization of many diseases [59]. The second is represented by targeted contrast agents. They are usually paramagnetic species glued to or part of specifically molecules, such as antibodies that are directed toward and taken up by specific molecular targets [60–62]. The third is represented by so-called “smart” contrast agents. Although they do not rely on selective targeting to achieve spatial specificity, they change their efficacy only in response to specific local molecular characteristics, such as the presence of specific proteinases, or changes in environmental pH [63–67]. The fourth is represented by labeled cells. They can be bound to or introduced into specific cell types such as T cells or stem cells, which then rely on the trafficking and recognition of the cells for their localization [68–71].

The above four types of MR contrast agents can be all used in different aspects of MRI molecular imaging. Furthermore, Ko et al. and Yang et al. [72, 73] reported the construction of PET/MRI dual-mode probe, which can perform PET and MRI imaging at the same time of the tumor. Kircher et al. [74] further developed a new triple-modality MRI-photoacoustic-Raman nanoparticle (MPR nanoparticle) to perform multimodal molecular imaging, and the three-mode molecular probe can obtain more accurate brain tumor resection by exploiting the complementary strengths of each modality.

Overall, magnetic resonance imaging is one of the most important molecular imaging research methods, which can be used for noninvasive monitoring and early diagnosis of diseases at the cellular and molecular level. In recent years, the study of magnetic resonance molecular imaging is increasing and is mainly used for cell tracking, angiogenesis, apoptosis, and in vivo tissue gene imaging. Although the techniques still have some problems that need an urgent solution, its unique advantages make its application prospect worthy of expectation in clinical medicine and basic research.

### 2.3. Radionuclide Molecular Imaging

Radionuclide imaging is one of the four major medical imaging techniques, and it is a radioactive marker in drug, when the body organs and tissue absorb and form radiation source in the body, and then, the nuclide detection device can be used to detect isotope in the process of decay on the rays, which constitute the image of radioactive isotopes in in vivo distribution density [75, 76]. In recent years, with the rapid development of molecular biology and nuclear medicine technology, the field of nuclear medicine has formed a new branch of nuclear medicine—molecular nuclear medicine [77]. SPECT and PET are advanced radionuclide molecular imaging techniques that are able to evaluate biochemical changes and levels of molecular targets within a living subject. Both techniques enable whole body imaging of molecular targets/processes with high sensitivity. SPECT is mainly used for whole body bone imaging [78–80], myocardial blood flow imaging [81–84], cerebral blood flow imaging [85–89], and thyroid imaging [90–95]. PET is mainly used to detect dynamic changes in the metabolic function of substances (or drugs) in the human body, and it widely used for the nervous system, the cardiovascular system, and the oncology [96–101].

The imaging agents used for PET are the basic elements for the human body, used to easily mark compounds and metabolites, and do not change their biological activity, so as to reflect the molecular level of physiological and biochemical processes of the human body, to achieve the purpose of early diagnosis and guidance of treatment. In clinical practice, PET that enables to locate, stage, and monitor cancer is mainly used to image tumors through the use of the 18F-labeled imaging agent [18F]-2-fluoro-2-deoxy-glucose ([18F]-FDG) [102–104]. For instance, Brenner et al. [105] reported that the predictive values of [18F]-FDG in primary staging in patients with newly diagnosed nonseminomatous germ cell tumor (NSGCT) clinical stage I/II. Kindred et al. [106] investigated glucose uptake asymmetries in leg muscles of patients with mild multiple sclerosis (MS) during walking with a glucose tracer (18F-FDG),and the result showed that [18F]-FDG uptake was significantly lower in the weaker knee flexors of patients with MS. Winther-Larsen et al. [107] evaluated whether changes in [18F]-FDG uptake evaluated early during erlotinib treatment predict survival in nonsmall-cell lung cancer (NSCLC) patients.

In addition to its clinical practicality, PET has extensive applications in the basic and preclinical researches. PET can be used to study basic physiological and molecular mechanisms of human diseases by using the appropriate radiolabeled-imaging agents [108]. Bretin et al. [109] reported that the biodistribution of the PET tracer over time can be determined in vivo. Moreover, PET can be employed for the evaluation of novel radiolabeled-PET imaging agents, biodistribution of novel pharmaceuticals in suitable animal models, and effectiveness of new therapies [30, 110]. For instance, Nanni et al. [111] studied small animal PET for the early detection of malignant masses in a xenograft murine model of human rhabdomyosarcoma and analyzed the metabolic behavior of this xenograft tumor over time.

SPECT imaging agents use energy between 85 and 500 Kev; radiographic tomography is a technique for projection reconstruction of faulty images, which are similar to X-ray and CT imaging. SPECT uses nuclides such as ^99m^Tc [112–114], and ^123^I [115, 116] through the emission of single gamma rays decay to obtain different energies. SPECT is one of the most commonly used nuclear medicine modalities in clinical practice [117]. Some examples of clinical use are the potential usefulness of ^99m^Tc-TRODAT-1 imaging in the evaluation of patients with early-stage Parkinson's disease [118], the therapeutic effects of ^111^In-DTPA-octreotide in tumors of various sizes [119], and the location and excision of the tumor [120]. Besides, small-animal SPECT is designed for imaging small animals specifically [121, 122], and it has been used for many preclinical studies. To name a few, Wang et al. [123] studied that the dynamics and feasibility of imaging nonsmall-cell lung cancer (NSCLC) apoptosis induced by paclitaxel treatment using ^99m^Tc-labeled C2A domain of synaptotagmin I in a mouse model. Moscaroli et al. [124] presented an ^111^In-radiolabeled FGF-2 derivative for noninvasive imaging in small animals deploying single-photon emission tomography (SPECT). Tang et al. [125] developed a radiolabeled tyrosine kinase inhibitor (TKI) for HER2-targeted breast cancer imaging.

Since biochemical changes always occur before anatomical changes in disease, both PET and SPECT have clear diagnostic strength over anatomical techniques such as classical CT and MRI. However, PET and SPECT have a key weakness, that is, the lack of an anatomical reference frame. This weakness may be eliminated through the combination of these instruments with either CT or MRI, producing a single scanner capable of accurately identifying molecular events with precise correlation to anatomical findings [126]. This method is known as “multimodality imaging,” in which two or more modalities are used in combination to develop their individual strengths and compensate for the weaknesses of each imaging system. For example, Glaus et al. [127] developed a novel nanoparticle-based dual-modality positron emission tomography/magnetic resonance imaging (PET/MRI) contrast agent; the probe produced strong MR and PET signals and were stabled in mouse serum for 24 h at 37°C. Chen et al. [128] synthetized folate-NOTA-Al^18^F radiotracer and examined its properties both in vitro and in vivo, for PET imaging of folate receptor-positive tumors. Xing et al. [129] built a dual-mode probe which uses RGD as a target probe and quantum point as the carrier. Then, this dual-mode probe is covered with PEG to improve water solubility and is linked to DOTA which chelates ^64^Cu. Thus, the nuclear medical imaging is associated with near-infrared imaging. Finally, the authors show that the PET signal is highly coincided with quantum dot near-infrared image.

So far, nuclear medicine molecular imaging technology is one of the widely used technologies in clinical molecular imaging technology and plays an important role in the study of personalized medical care due to its unique technology [130–133]. PET and SPECT are not only powerful tools for basic medicine and pharmacy but also the best tools for detecting and guiding the treatment of various diseases and tumors [110, 134, 135]. They contribute to developing treatment programs on the tumor and other diseases, which are made by clinicians more scientific, more comprehensive, and more reasonable. Their application will have a profound impact on clinical practice.

### 2.4. Ultrasound Molecular Imaging

Ultrasound imaging, like MRI and CT, has been used as a morphological imaging modality. Medical ultrasound imaging is a unique imaging modality that exploits the properties and behavior of high-frequency sound waves as they travel through biological tissue, and it can be used both for diagnostic imaging and as a therapeutic tool. Compared with traditional imaging techniques such as radionuclide imaging and optical imaging, ultrasound imaging has some advantages such as economy, convenience, and real-time imaging [136, 137]. Furthermore, ultrasound molecular imaging of contrast agents which combine with the target organ can be used as a carrier for therapeutic drugs or genes, so as to achieve a multiplier effect [138–140].

Traditional ultrasound contrast agents, that are a few micrometers in diameter and in the terms of gas-filled microbubbles, are often coated with lipids or biopolymers, and they are available for enhancing the reflection signal-to-noise ratio for blood [141]. These contrast agents have provided useful imaging data, but they do not enable imaging of specific molecular events. However, by attaching certain antibodies [142–144], peptides [145, 146], or other targeting moieties [147] to the surface of microbubbles, those particles can target specific biochemical processes to achieve ultrasound molecular imaging. Ultrasound molecular imaging that uses *in vivo* simulation of immunohistochemistry or *in situ* hybridization techniques targets biomolecules to highlight the pathological changes of diseased tissue. Thus, it can visualize the real pathogenesis and significantly improve the sensitivity and accuracy of imaging diagnosis. These aspects are actually the current clinical research central issues.

At present, targeted microbubbles are being used in preclinical investigations of both inflammation and angiogenesis. For example, microbubble shells have been attached to endothelial cell adhesion molecules for visualization of P-selectin, supplying foresight on molecular aspects of inflammation [148]. Deshpande et al. [149] showed that P-selectin-targeted microbubbles (MBs) can be used to monitor the expression of this molecule as a marker of inflammation in a murine model of inflammatory bowel disease (IBD). In order to target vascular endothelial growth factor receptor-2 (VEGFR2), Willmann et al. [150] constructed anti-VEGFR2 antibodies attached to microbubbles. After using either targeted microbubbles or control microbubbles in tumor-bearing nude mice, ultrasound imaging studies can be performed. Compared with studies using only control microbubbles, imaging results demonstrated a significantly higher average intensity in images from studies using targeted microbubbles. Liu et al. [145] developed endothelial-targeted microbubbles (MBs) and employed targeted microbubble-enhanced ultrasound (US) imaging to assess the endothelial expression levels in neovasculature for noninvasive assessment of colorectal tumor angiogenesis.

With the emergence of ultrasound molecular imaging, the early diagnosis and specific treatment of malignant tumors gained some research achievements. Kim et al. [151] used ink-containing PLGA polymer microbubbles to achieve both enhanced optical and ultrasound imaging of breast cancer with a depth of less than 18 mm. Cochran et al. [152] study showed that injecting paclitaxel microbubbles can effectively avoid acute poisoning reaction caused by direct injection of paclitaxel, thus significantly reducing paclitaxel side effects and effectively inhibiting tumor growth. Wang et al. [153] reported that integrin-targeted nanoparticle gene vector can specifically act on vascular endothelial cells of mice tumors, thus inducing tumor regression. Liu et al. [154] prepared perfluoropentane nanodroplets modified by folate and encapsulated by lipid membrane (FA-NDs) and investigated the nanodroplets stability in different temperature and its target performance of SKOV3 tumor cells in vitro and in vivo. Hu et al. [155] verified that tumor over expression of SHP2 and other protein tyrosine phosphatases regulated several cellular processes and contributed to tumorigenesis, which could be introduced to ultrasound molecular imaging for differentiating normal from malignant thyroid diagnostic nodes.

In the near future, ultrasound molecular imaging is expected to pass from a preclinical modality to a fully clinically useful technique through the use of different clinically translatable instrumentation, such as endoscopes and novel US-compatible imaging agents that are able to exudate [156, 157]. Among the agent-based molecular imaging techniques, targeted ultrasound imaging has promising huge developments.

### 2.5. Optical Molecular Imaging

Optical imaging is a method of obtaining biological information by using optical detection means combined with optical detection molecules to imaging cells or tissues or even organisms. If the biological optical imaging is limited to the visible and near-infrared range, different biological optical imaging methods can be divided into fluorescence imaging, bioluminescence imaging, photoacoustic imaging, and optical tomography. Nowadays, molecular imaging becomes more popular and is combined with classical optical imaging techniques.

Fluorescence imaging technology is marked with a fluorescent report group including inorganic materials, such as upconversion, quantum dots, and other organic materials, such as green fluorescent protein, red fluorescent protein, or fluorescent dye. It uses excitation light to make the report group reach a higher level of molecular level and then emit a longer wavelength visible light to form biological light source in vivo and detect it. At present, common fluorescent groups include various small molecule fluorescent dyes, green fluorescent protein, and red fluorescent protein. In recent years, fluorescence technology has been extensively used in the study of molecular biology and the metabolism of small molecules in small molecules. There is a rapidly expanding list of fluorescent agents which includes near-IR Cy 5.5, turnip green, quantum dots (QDots), the Alexa dye series, and all kinds of fluorescent proteins. Besides, lanthanide-based imaging agents were added to this list [158]. There are numerous strengths of these agents which are better than the aforementioned dyes and proteins. They have narrow, nonoverlapping emission bands, long luminescence lifetimes, and allow multiplexed quantitative measurements of the intracellular analysis concentrations [158, 159]. Fluorescence molecular tomography (FMT) has been applied to visualize and quantitate a variety of cellular and molecular events. In opposition to planar fluorescence imaging, FMT can produce quantitative information and allows imaging at greater depths, up to several centimeters [10]. In 2009, Hyde et al. [160] first used FMT/CT dual-mode imaging to observe the distribution of the focal region of the brain in the mouse model of Alzheimer's mice. Lin et al. [161] developed a FMT/MRI fusion imaging system for small animal imaging, which verified the accuracy of FMT imaging provided by the anatomical structure provided by MRI.

Bioluminescence imaging technology uses luciferase gene to label cells or DNA and exploits sensitive optical detection instrument to directly monitor cell activity and gene behavior in living subjects. This technique has these following advantages: (1) noninvasive, (2) continuous repeated detection, (3) fast real-time scanning imaging, and (4) high sensitivity. Bioluminescence imaging has been used to study numerous enigmatic protein-protein interactions. One such study uses a firefly luciferase-based protein fragment complementation assay to visualize luciferase-expressing bone marrow cells in brain inflammation in living mice [162]. In 2006, Wang et al. [163] built a separate BLT/CT dual-mode imaging system, BLT data acquisition system to the mice in the imaging process, and then saved the mouse position and posture, and the acquisition of CT data, finally anatomical information obtained from CT into BLT reconstruction, so as to improve the accuracy of BLT imaging.

Photoacoustic imaging (PA) by using optical absorption and transformation between the tissues of the light and sound energy is a nondestructive imaging method developed in recent years. It combines the high penetration characteristics of pure optical imaging and high contrast characteristics by light into the ultrasound, and it can provide tissue imaging with high resolution and high contrast. Based on the technology of photoacoustic effect of time-domain photoacoustic spectrum, it partially overcomes the effect of strong scattering in the optical transmission in the organization when optical and acoustic are organically combined. Therefore, photoacoustic technology has well biological penetration, the characteristics of high resolution, and no side effects. Its main application direction can effectively carry out biological tissue structure and function imaging, providing an important means for studying the morphological structure, physiological characteristics, pathological characteristics, and metabolic function of biological tissue. For example, Ding et al. [164] developed a novel contrast agent where the surface of superparamagnetic iron oxide (SPIO) nanoparticles is functionalized with a bladder cancer-specific fluorescein isothiocyanate- (FITC-) labeled cell penetrating peptide- (CPP-) polyarginine peptides (R11) for active targeting and imaging. The results indicate great potential of SPIO-R11 as a contrast agent to target bladder cancer for diagnostic and therapeutic applications.

At present, there are still some defects in the living organism imaging system. Many in vivo optical imaging are also just stay in the phantom and small animal experimental stage, has not yet entered into clinical application, and need a further improvement in many aspects. It is an important task for the future to find new high quantum efficiency fluorophores, improve the reconstruction algorithm and image resolution, and expand the new optical imaging technology. In vivo bioluminescent imaging technology has become an indispensable tool in the research of nuclear small-animal models. It studies the pathological process, drug development, and drug efficacy from a unique perspective. In fact, the biological optical imaging technology has had a significant impact on the basic and applied medical research.

## 3. Application of Multimodal Molecular Imaging

Multimodal molecular imaging combines two kinds or more detection technologies to form a new way of imaging, which is convenient for obtaining some further information in diagnosis, treatment, and monitoring. At present, multimodal molecular imaging has been widely used to optimize medical research and clinical practice. In practice, multimodal molecular imaging has been helpful for cardiovascular diseases [165, 166], neuropsychiatric diseases [167–170], and other clinical diseases [168, 171–175]. In addition, it can significantly enhance the positioning of the tumor border and effectively guide the surgical resection of the tumor [74, 176, 177].

A few specific examples are as follows: (1) cardiovascular diseases: Yoo [178] presented multimodal intravascular optical imaging combining optical coherence tomography and fluorescence lifetime imaging. It can provide new opportunities to investigate vascular pathobiology and diagnose cardiovascular disease, by simultaneously visualizing plaque morphology and biochemical composition; (2) neuropsychiatric diseases: Voss et al. [179] used multimodal functional-imaging technology to study a patient with marked neurological recovery after cranioplasty, and the results suggested resting-state networks and auditory responses obtained with functional MRI and cerebral metabolism obtained with PET before and after cranioplasty revealed significant functional changes that were correlated with the subject's neurological recovery. Wang [180] studied the multimodal imaging investigation of brain mechanisms in neuropsychiatric disorders, emphasizing on the research questions of whether and how neurochemistry is associated with brain anatomical structures and brain functions; and (3) other clinical diseases: Tang et al. [175] developed a novel multimodal video endoscope and evaluated its usefulness for the early detection of gastric neoplastic lesions; the imaging platform is a modified upper GI endoscope capable of white-light imaging (WLI), wide field vital-dye fluorescence imaging (VFI), and high-resolution microendoscopy (HRME) in a single endoscopic insertion.

In summary, multimodal molecular imaging has a bright future. The development of this field will bring a major breakthrough in medical imaging and molecular biology. Although molecular imaging remains at its initial stage, a broader space for further developments is still possible.
